# Relationship between Atrial Tachyarrhythmias and Intrathoracic Impedance in Patients with a Pacemaker and Preserved Ejection Fraction

**DOI:** 10.3390/jcm9010105

**Published:** 2019-12-31

**Authors:** Min-Tsun Liao, Chun-Kai Chen, Ting-Tse Lin, Li-Ying Cheng, Hong-Wen Ting, Rou-Fang Wang, Zhi-Jun Liu, Yen-Bin Liu

**Affiliations:** 1 Division of Cardiology, Department of Medicine, National Taiwan University Hospital Hsinchu Branch, Hsinchu 300, Taiwan; liaomintsun@gmail.com (M.-T.L.); ineosky@gmail.com (C.-K.C.); aesculapius0214@gmail.com (T.-T.L.); chengliying2@gmail.com (L.-Y.C.); sharon3458@gmail.com (H.-W.T.); may910702@gmail.com (R.-F.W.); miss930906@gmail.com (Z.-J.L.); 2 College of Medicine, National Taiwan University, Taipei 100, Taiwan; 3 Division of Cardiology, Department of Medicine, National Taiwan University Hospital, Taipei 100, Taiwan

**Keywords:** OptiVol fluid index, intrathoracic impedance, atrial tachyarrhythmias

## Abstract

Atrial fibrillation (AF) is responsible for significant morbidity and mortality in patients with heart failure (HF). Modern pacemakers provide an index of intrathoracic fluid status (OptiVol fluid index—OVFI) by measuring daily intrathoracic impedance. This study aimed to determine whether OVFI is associated with increased atrial tachycardia/fibrillation (AT/AF) events in patients with a preserved ejection fraction (EF). We retrospectively reviewed data from patients with Medtronic Advisa pacemakers between 2012 and 2014 in our hospital. The association and temporal relationship between OVFI and AT/AF events were determined. A total of 150 patients with 211 follow-up visits (mean 1.4 visits per patient) were evaluated. The device-detected AT/AF prevalence was 47%. Device-measured OVFI ≥ 20 Ω-days was significantly associated with the onset of AT/AF ≥ 4 h. OVFI threshold crossing preceded AT/AF events in 55.1% of cases, followed by AT/AF events in only 18.7%. Fluid overload represented by OVFI may trigger AT/AF episodes in patients with a preserved EF more often than that previously reported in patients with a reduced EF. Our findings support the view that worsening pulmonary congestion is associated with increased AT/AF frequency and suggests that fluid overload could trigger and perpetuate AT/AF events in patients with a preserved EF.

## 1. Introduction

Atrial fibrillation (AF) is responsible for significant morbidity and mortality in patients with heart failure (HF) with a reduced ejection fraction (HFrEF) or preserved ejection fraction (HFpEF) [1,2]. It is widely accepted that the coexistence of HF and AF carries a worse prognosis than either alone. There is a reciprocal relationship between HF and AF in which HF predisposes individuals to AF and AF worsens HF [3], but most data are from patients with HFrEF. The prevalence of AF increases with increasing ejection fraction (EF) in patients with HF [3,4]. Zafrir et al. [4] reported that AF was independently associated with poorer cardiovascular outcomes in patients with HFpEF than in those with HFrEF, suggesting that AF may be correlated with different pathogeneses in HFrEF and HFpEF. Modern cardiac implantable electronic devices (CIEDs) are capable of continuously monitoring atrial tachyarrhythmia (AT)/AF and measuring daily intrathoracic impedance (ITI). The OptiVol Fluid index (OVFI; Medtronic, Minneapolis, MN, USA), a fluid status algorithm calculated from ITI trends, was designed to aid in the diagnosis of fluid overload and pulmonary congestion before HF worsening [5,6]. The relationship between AT/AF and fluid overload is complex and has been only partly described, particularly in patients with HFpEF. The broad adoption of CIEDs has provided a unique platform to develop ancillary sensor technologies to better understand longitudinal physiological alterations in AT/AF and fluid overload. Multiple studies have attempted to investigate the association between decreased thoracic impedance quantified by the OVFI and AT/AF [7,8]. However, most patients in those clinical studies related to OVFI have HFrEF [9]. Thus, whether ITI monitoring could predict AT/AF events in patients with HFpEF with a similar threshold of ITI trends as patients with HFrEF remains unclear. This study aimed to examine the association between periods of increasing OVFI and the occurrence of CIED-documented AT/AF in patients with a pacemaker and preserved left ventricular ejection fraction (LVEF). We further sought to determine the temporal relationship between AT/AF events and fluid overload in our patient population.

## 2. Materials and Methods

### 2.1. Design

This study included a retrospective analysis of data from patients with a Medtronic Advisa pacemaker (OptiVol build-in) capable of measuring intrathoracic electric impedance and an LVEF ≥ 50% between 2012 and 2014 in our hospital. The indications of pacemaker implantation were sick sinus syndrome (SSS) and atrioventricular (AV) block. Patients were excluded if 100% AT/AF was found. This study was approved by the Institutional Review Board of National Taiwan University Hospital.

### 2.2. ITI Measurement

Determination of the ITI-derived fluid index (OptiVol™, OVFI, Medtronic Inc., Minneapolis, MN, USA) was described previously [8]. Briefly, ITI is measured each day directly between the right ventricular lead and the device metal case to determine an average daily impedance value. The reference impedance trend is determined from the trend in daily impedance values. The calculated reference impedance represents the expected or normal impedance that may vary over time within patients. The OVFI increases when the daily impedance consistently falls below the reference impedance. The OVFI returns to zero when the daily impedance value trends at or above the reference impedance. The amplitude of the OVFI is determined by both the magnitude of the impedance reduction relative to the reference impedance level (Ω) as well as the duration of the relative reduction in daily impedance (days). The OVFI can be compared to the programmed threshold parameter with a nominal value of Ω-days. A fluid index value at or above the threshold (i.e., OVFI threshold crossing) may be an indicator of thoracic fluid overload.

### 2.3. Endpoints

The first purpose of this study was to evaluate the relationship between OVFI (as a surrogate of fluid overload and pulmonary congestion) and AT/AF frequency (defined as at least one episode of AT/AF more than 4 h) and burden (defined as the percentage of AT/AF time since last visit). The cut-off value of 4 h used in the setting of our study was due to the classification of AT/AF duration by Medtronic Advisa. The secondary purpose of this study was to access the temporal sequence between OVFI and AT/AF episodes. The cut-off value of the duration for threshold crossing the OVFI preceded or followed by device-detected AT/AF more than 4 h was 30 days. A previous study demonstrated that a threshold-crossing OVFI for > 30 days was strongly indicative (5.5 times) of decompensated heart failure requiring hospitalization [10]. We used 30 days as the cut-off value because 30 days was also better for the manual determination for the temporal sequence between OVFI and AT/AF episodes. The temporal relationship was reviewed by two experienced nurse practitioners (LYC and HWD) and two nurses (ZJL and RFW), who then discussed the classification with the cardiologist physician (MTL). Daily fluid index measurements and values of time in device-detected AT/AF episodes were used to identify patients with OVFI threshold crossings with or without AT/AF. Different thresholds of OVFI were also evaluated.

### 2.4. Follow-Up

Data of each visit were collected from interrogation recordings of pacemakers including AT/AF duration, AT/AF frequency, AT/AF burden, and OVFI value. All records were reviewed independently by 3 cardiac electrophysiologists (MTL, TTL, and YBL). AT/AF events were defined as episodes of duration > 5 beats and atrial rate ≥ 150–171 bpm. Different cutoff values were used according to the patients and attending physician. AT/AF was diagnosed as an irregularly irregular atrial rate ≥ 150–171 bpm recorded by intra-atrial electrograms. An echocardiography system (ie33, Philip, Andover, MA) equipped with an S5 transducer was used for the evaluation of cardiac function and structure. Two-dimensional, M-mode, and Doppler ultrasound recordings were included to measure the left ventricular dimension, septum and posterior wall thickness, left atrial dimensions, and LVEF. Transmitral flow velocity with E wave, A wave, and E wave deceleration times were measured.

### 2.5. Statistical Analysis

Descriptive statistics are recorded as mean ± standard deviation for normally distributed continuous variables and compared using means of Student’s t-test and analysis of variance. Categorical data are reported as percentages and were compared using the Chi-square test or Fisher’s exact test. The receiver operating characteristic (ROC) curve was analyzed to evaluate the sensitivity and specificity of OVFI in predicting AT/AF episodes more than 4 h. Generalized estimating equations (GEEs) with an autoregressive correlation structure were used to analyze AHRE frequency (binary outcome, >4 h) to account for possible correlations between visits within a patient. Univariate and adjusted analyses were performed. The following covariates were considered in the adjusted GEE models: age, sex, prior AF, prior HF, use of anti-arrhythmia agents and beta-blockers, and mitral valve (MV) E wave. Backward selection was used and covariates with probability values < 0.1 were retained in the model. Probability values < 0.05 were considered statistically significant. The GEE method developed by Liang and Zeger [11] was used to produce regression estimates when analyzing repeated measures with non-normal response variables. Statistical analysis was performed using SPSS version 22.0 for Windows (SPSS Inc., Chicago, IL, USA).

## 3. Results

### 3.1. Study Population and Echocardiography

Findings in 150 patients with 211 follow-up visits (mean, 1.4 visits per patient) were evaluated. The median follow-up time among all subjects was 4.70 months. There were 68 men and 82 women with a mean age of 75.4 ± 11.3 years old. Patients with both SSS and AV block were 72.7% and 27.3%, respectively. The basic clinical characteristics of the enrolled patients were based on a review of the medical records with admission and discharge diagnoses in our hospital. The systemic diseases of these patients included congestive heart failure (11.3%), hypertension (78.0%), diabetes mellitus (29.3%), cerebrovascular accident (CVA), transient ischemic attack (TIA) (11.3%), coronary artery disease (CAD) (22.0%), peripheral artery disease (PAD) (2.0%), and prior AF (42.0%). The patients’ baseline characteristics are shown in Table 1.

The mean LVEF was 68.0% ± 6.6% measured by transthoracic echocardiography. The LVMI was 143.3 ± 43.5 g/m^2^. The LA dimension was 38.9 ± 6.7 mm. The mitral valve E wave and A wave were 82.7 ± 27.5 and 86.8 ± 25.5 cm/s, respectively. The echocardiographic parameters are shown in Table 2.

### 3.2. Prevalence of AT/AF and Heart Failure Hospitalization

During the follow-up period, 47% of the patients had at least one episode of AT/AF detected by pacemakers, including paroxysmal AT/AF (35.8%) and persistent AT/AF (11.3%). Paroxysmal AT/AF was defined as continuous AT/AF for less than 7 days, while persistent AT/AF was defined as AT/AF lasting longer than 7 days [12]. There was only one patient hospitalized for heart failure, and two others died during the study period, including one for end-stage colon cancer, and the other for sudden cardiac death.

### 3.3. ROC Analysis in Different Cut-Off Values of OVFI for AT/AF Episodes

**Figure 1:**
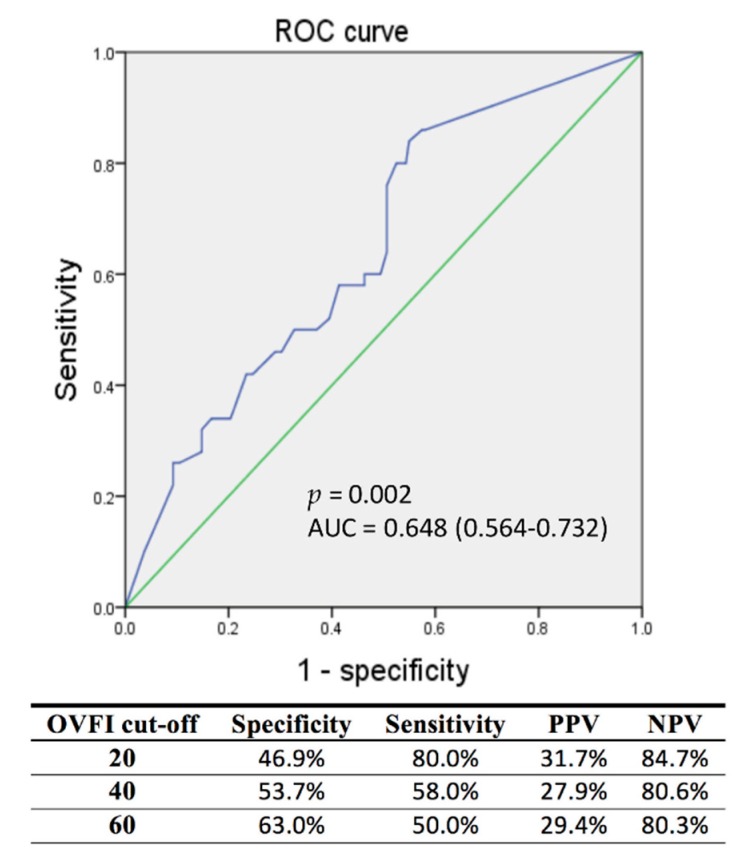
ROC analysis to determine sensitivity and specificity of OVFI for AT/AF episodes more than 4 h. AF, atrial fibrillation; AT, atrial tachyarrhythmia; AUC, area under the curve; NPV, negative predictive value; OVFI, OptiVol fluid index; PPV, positive predictive value; ROC, receiver operating characteristic.

In ROC analysis, OVFI could predict AT/AF of more than 4 h significantly (*p* = 0.002, areas under the ROC curve: 0.648, 0.564–0.732). The sensitivity, specificity, positive predictive value and negative predictive value were 80.0%, 46.9%, 31.7%, and 84.7%; 58.0%, 53.7%, 27.9%, and 80.6%; 50.0%, 63.0%, 29.4%, and 80.3% with a cutoff of 20, 40, and 60 Ω-days, respectively (Figure 1).

### 3.4. AT/AF and Patients’ Baseline Characteristics

Basic characteristics associated with device-detected AT/AF were analyzed by the GEE model with repeated measurements. As expected, all prior AF episodes were significantly associated with AT/AF frequency during follow-up (odds ratio (OR), 15.484; 95% confidence interval (CI), 5.408–44.339; *p* < 0.001). Anti-arrhythmia drugs and beta-blockers were also significantly associated with AT/AF frequency (OR, 17.176; 95% CI, 5.705–51.715, *p* < 0.001; and OR, 2.343, 95% CI, 1.091–5.031, *p* = 0.029, respectively). Other basic characteristics including congestive heart failure, hypertension, age, diabetes mellitus, CVA or TIA, CAD, PAD, or LVEF were not associated with increased AT/AF frequency by univariate analysis of the GEE model. The results are shown in Table 3.

### 3.5. OVFI Versus AT/AF Frequency

In the univariate GEE model, AT/AF frequency (percentage of patient visits with at least one episode of AT/AF more than 4 h since previous device interrogation) was greater in the OVFI ≥ 20 versus OVFI < 20 groups (OR, 1.975; 95% CI, 1.020–3.825; *p* = 0.044) and OVFI ≥ 40 versus OVFI < 40 (OR, 2.469; 95% CI, 1.202–5.071, *p* = 0.014). At an OVID cut-off of 60, there was no association with increased AT/AF frequency (OR, 1.567; 95% CI, 0.654–3.756, *p* = 0.314). The results are shown in Table 3.

In a GEE model adjusted for age, sex, prior AF, prior HF, use of anti-arrhythmic agents and beta-blockers, and MV E wave, AT/AF frequency was greater in OVFI ≥ 20 versus OVFI < 20 (*p* = 0.035). The other independent covariates were prior AF (*p* < 0.001) and the use of anti-arrhythmic agents (*p* < 0.001). There was no significant association with increased AT/AF frequency between OVFI ≥ 40 versus OVFI < 40 and OVFI ≥ 60 versus OVFI < 60 (*p* = 0.063 and *p* = 0.233, respectively). The results are shown in Table 3.

### 3.6. OVFI Versus AT/AF Burden

There were 92 and 119 visits with AT/AF burdens of 9.0% ± 22.3% and 7.4% ± 21.8% in the OVFI < 20 and OVFI ≥ 20 groups, respectively. Neither number of visits nor AT/AF burden differed significantly between the OVFI < 20 and OVFI ≥ 20 groups. The number of AT/AF episodes ≥ 4 h since previous device interrogation was greater in the OVFI ≥ 20 versus OVFI < 20 group (*p* = 0.015). However, such increases in AT/AF frequency were not demonstrated in AT/AF episodes of less than 4 h. The results are shown in Table 4.

### 3.7. Temporal Sequence between OVFI and AT/AF Frequency

**Figure 2:**
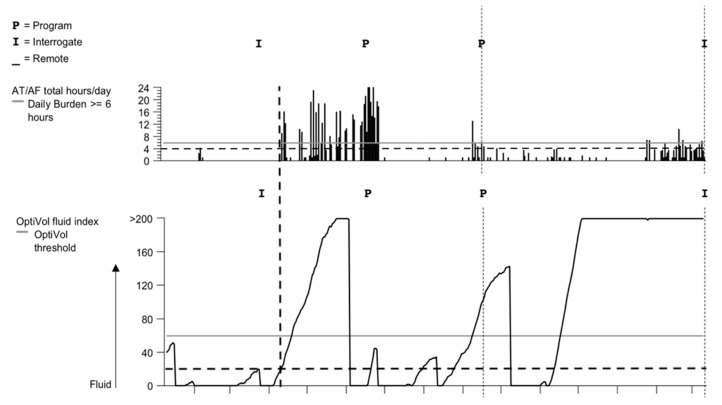
A representative tracing of the OptiVol fluid index (OVFI)-capable pacemaker that illustrates the AT/AF burst occurring after an OVFI increase to above the threshold of 20. AF, atrial fibrillation; AT, atrial tachyarrhythmia.

The temporal sequence between AT/AF episodes and threshold crossing of OVFI ≥ 20 was evaluated. The 30-day interval was used as the cut-off value of the duration for threshold crossing of OVFI preceded or followed by device-detected AT/AF episodes more than 4 h by manual counting according to the tracing graph. There were three possible situations: AT/AF episodes appearing before (AT/AF resulted in fluid overload), after (fluid overload triggered AT/AF), or simultaneously with a threshold crossing of OVFI ≥ 20 Ω-days. The threshold crossing of OVFI ≥ 20 preceding AT/AF episodes was the most common finding in 55.1% (609/1105) of incidences. The threshold crossing of OVFI ≥ 20 followed AT/AF episodes more than 4 h in 18.7% (207/1105) and was simultaneous or indeterminate in the other 26.2% (289/1105). A representative tracing demonstrates the threshold crossing of OVFI ≥ 20 before AT/AF episodes in Figure 2.

## 4. Discussion

Our study revealed that device-measured decreased ITI was statistically significantly associated with the onset of AT/AF episodes ≥ 4 h in CIED patients with a preserved EF. Instead of OVFI ≥ 60 Ω-days as the crossing threshold, which was mainly for patients with HFrEF, there was an association between OVFI ≥ 20 Ω-days and AT/AF frequency in our patients with a preserved EF. OVFI threshold-crossing preceded AT/AF events in 55.1% of incidences, followed by AT/AF events in only 18.7%. Fluid overload, represented by OVFI, may trigger AT/AF episodes in patients with a preserved EF more often than previously suspected in patients with HFrEF.

Previous studies of OVFI mainly included patients with HFrEF. The main reason is that OVFI-enabled devices were mainly used in patients with implantable cardioverter-defibrillators (ICD) or cardiac resynchronization therapy (CRT). Many studies have reported that decreases in device-measured ITI could be a useful predictor of impending clinically significant fluid overload and hospitalization due to HF exacerbation [13]. A retrospective study that analyzed a cohort of 21,217 patients with ICD or CRT found that patients with a threshold crossing of ITI detected by CIED were at an increased risk (by 2.15 times) of age- and sex-adjusted mortality [14]. HF and consequent fluid overload are well-known risk factors for both AT/AF and ventricular tachyarrhythmia/fibrillation (VT/VF). Acute increases in filling pressure can lead to electrical instability caused by changes in action potential duration and refractoriness as well as the development of afterdepolarizations [15,16,17]. Neurohormonal activation, electrolyte disturbances, and myocardial ischemia during acute HF may also exert proarrhythmic influences in the myocardium [18]. OVFI values calculated from ITI trends have been validated to inversely correlate with pulmonary capillary wedge pressure and fluid balance and with changes in N-terminal pro-hormone brain natriuretic peptide [8,12]. Multiple studies have investigated the association between decreased ITI quantified by the OVFI and both AT/AF and VT/VF in patients with HFrEF [8,19]. A meta-analysis summarizing the published evidence of the association between OVFI and development of AT/AF and VT/VF reported a statistically significant association between increased OVFI and AT/AF and VT/VF onset in patients with HFrEF. The pooled odds ratio for OVFI threshold crossing was 1.56 (95% CI, 1.35–1.81) for VT/VF and 1.8 (95% CI, 1.43–2.27) for AT/AF [20]. The results of the present study are consistent with those of prior studies of patients with HFrEF. The threshold crossing of device-measured OVFI was significantly associated with AT/AF events in CIED patients with a preserved EF.

Unlike previous studies, our study mainly aimed to examine patients with a preserved EF. Patients with SSS have a higher chance of AT/AF detection [21], and their LVEF was usually preserved. This provides a good opportunity to assess the relationship between OVFI and AT/AF events in patients with a preserved LVEF.

The OVFI threshold crossing associated with AT/AF occurrence is 20 Ω-days in our study instead of 60 Ω-days in patients with HFrEF. The determination of the OVFI is based on the trend of daily impedance value. Reference impedance represents the expected or normal impedance trend in patients with a preserved EF may vary over time differently from those with a reduced EF. The differences in the baseline impedance trend may affect the OVFI crossing threshold to detect the fluid overload in patients with a preserved EF. Meanwhile, in our study, the association between OVFI and AT/AF was demonstrated in cases of AT/AF episodes ≥ 4 h but not in episodes < 4 h. This finding may be affected by the false-positive or -negative rates while using atrial high rate episodes (AHREs) to diagnose AT/AF, especially when the duration of AHRE is brief. In the ASSERT study, 17.3% of the AHRE longer than 6 min were false positives. The rate of false positives was reduced to 3.3% when a longer threshold of 6 h was used for AHREs, suggesting that the longer the duration, the lower the number of false-positive detections [22]. The association between OVFI and AT/AF episodes ≥ 4 h also suggests that fluid overload may be important to AT/AF triggers and perpetuation. Electrical remodeling and abnormal calcium handling after fluid overload could promote AT/AF onset and make AT/AF last longer [23].

The temporal relationship between OVFI and AT/AF events in patients with a preserved EF differed from that of previous reports of patients with a reduced EF. Our study found that a threshold crossing of OVFI preceded AT/AF events ≥ 4 h in 55.1% of events and followed AT/AF events in only 18.7%. Previous studies suggested a threshold crossing of OVFI is more likely to occur after the onset of AT/AF events rather than preceding the onset of a persistent AT/AF in patients with reduced EF. In patients with HFrEF, neurohormonal activation and hemodynamic mechanisms were emphasized to perpetuate the vicious HF–AF cycle. In contrast, systemic inflammation and shared cardiovascular risk factors (such as aging, hypertension, and obesity) play more important roles in AF and HFpEF. The potential differences in the pathogenesis of AF between HFrEF and HFpEF indicate that fluid overload may be responsible for AT/AF in different ways considering the temporal relationship between OVFI and AT/AF events in our study. Besides, using 20 Ω-days instead of 60 Ω-days as the crossing threshold of OVFI may also alter the temporal relationship between OVFI and AT/AF events. If the crossing threshold of OVFI ≥ 20 Ω-d had been detected earlier, diuretics could have been used earlier to prevent further fluid overload, or anti-arrhythmic drugs could have been used to prevent AT/AF occurrence. However, setting the threshold downward may increase the possibility of a false positive. Our study revealed that there is a relatively low specificity and positive predictive value of OVFI for AT/AF events. According to our results, there will be an AT/AF event ≥ 4 h in the next 1 month for every two threshold crossings of OVFI. Further research may need to combine other parameters and reduce the rate of false positives for this approach to be more suitable for clinical decision making.

There are several limitations to our study. First, it was a retrospective study and we reviewed only the pacemaker recordings. OVFI-guided therapy could not be performed. There may be selection bias due to the source population and missing data from irregular follow-ups. This may have an underdiagnosis of clinical characteristics. Additionally, the cardiac biomarkers such as high-sensitivity troponin and natriuretic peptide were not available in our study. However, current evidence only offers a class IIb recommendation to reduce stroke and bleeding risks in patients with atrial fibrillation rather than predicting the occurrence of atrial fibrillation [12]. In our study, the results of ROC showed low discrimination because of the low areas under the ROC curve. Therefore, other unmeasured co-variates may also affect the explanatory power of OVFI ≥ 20 Ω-d. Previous research has also shown that pneumonia, pleural effusion, and pneumothorax can cause false positive of OVFI alarms [24]. Second, this study only provides data of OVFI and AT/AF frequency, but the effect on hard endpoints such as heart failure hospitalization, morbidity, and mortality cannot be analyzed because of rare events. OVFI threshold crossing is a surrogate with fluid overload. Whether OVFI threshold crossing can predict heart failure hospitalization in patients with a preserved EF remained to be investigated. Due to the small sample size and low event rate of heart failure hospitalization in our study, the association between OVFI and heart failure hospitalization could be not demonstrated. Further long-term follow-up studies with a larger sample size are needed to evaluate the clinical impact of OVFI on AT/AF frequency and hospitalization due to heart failure. Third, the temporal relationship was evaluated by manual determination, and this may have created an artificial error with the different classifications. However, two experienced nurse practitioners and two nurses analyzed their respective calculations and then discussed the classifications with the cardiologist, which can reduce the human error. Fourth, our study demonstrated only a modest temporal relationship between OVFI and AT/AF events. Despite our study demonstrating that a threshold crossing of OVFI precedes AT/AF events in most cases, unlike reports of prior studies in patients with reduced EF, the relationship between OVFI and AT/AF events may be mutually causal. Further prospective studies are needed to distinguish the true causal relationship and clinical impact between OVFI and AT/AF.

## 5. Conclusions

Our findings not only support the view that worsening pulmonary congestion is associated with increased AT/AF frequency, but they also suggest that fluid overload may trigger and perpetuate AT/AF events in patients with a preserved EF. However, further research needs to investigate whether a combination of OVFI thresholds and other clinical information could improve the diagnostic accuracy of fluid overload and its association with AT/AF events.

## Figures and Tables

**Table 1:** Baseline characteristics of study participants.

Clinical Characteristic	*N* = 150
**Demographic and medical history**	
Age (years)	75.4 ± 11.3
BMI	24.8 ± 3.7
Male sex	68 (45.3%)
Congestive heart failure	17 (11.3%)
Hypertension	117 (78.0%)
Diabetes mellitus	44 (29.3%)
CVA or TIA	17 (11.3%)
Coronary artery disease	33 (22.0%)
Peripheral artery disease	3 (2.0%)
SSS	109 (72.7%)
Prior AF	63 (42.0%)
**Pharmacologic therapy**	
Beta-blockers	68 (45.3%)
ACEi/ARB	89 (59.3%)
Calcium channel blockers	68 (45.3%)
Diuretics	62 (41.3%)
Antiarrhythmic agents	79 (46.7%)

Values are expressed as mean (SD). ACEi, angiotensin-converting enzyme inhibitor; AF, atrial fibrillation; ARB, angiotensin receptor blockers; AVB, atrioventricular block; BMI, body mass index; CVA, cerebrovascular accident; SSS, sick sinus syndrome; TIA, transient ischemic attack.

**Table 2:** Echocardiographic parameters of the study population.

Echocardiographic Parameters	*N* = 150
IVST, mm	11.4 ± 1.9
LVPWT, mm	10.8 ± 1.7
LVEDD, mm	46.8 ± 5.4
LVESD, mm	28.4 ± 4.4
LVEDV, mL	100.8 ± 28.1
LVESV, mL	30.9 ± 11.1
LVEF, %	68.0 ± 6.6
LVM, g	241.5 ± 74.7
LVMI, g/m^2^	143.3 ± 43.5
LA dimension, mm	38.9 ± 6.7
MV E, cm/s	82.7 ± 27.5
MV A, cm/s	86.8 ± 25.5
MV deceleration time, s	0.22 ± 0.07

Values are expressed as mean (SD). IVST, interventricular septal thickness; LA, left atrium; LVEDD, left ventricular end-diastolic diameter; LVEDV, left ventricular end-diastolic volume; LVEF, left ventricular ejection fraction; LVESD, left ventricular end-systolic diameter; LVESV, left ventricular end-systolic volume; LVM, left ventricular mass; LVMI, left ventricular mass index; LVPWT, left ventricular posterior wall thickness; MV, mitral valve.

**Table 3:** Factors associated with AT/AF episodes more than 4 h.

Clinical Characteristic	OR	95% CI		*p* Value
**Demographic and medical history**				
Age	0.982	0.951	1.015	0.291
BMI	1.048	0.926	1.186	0.455
Male sex	1.344	0.632	2.861	0.443
Congestive heart failure	1.019	0.353	2.940	0.973
Hypertension	1.071	0.428	2.676	0.884
Diabetes mellitus	1.050	0.456	2.417	0.909
CVA or TIA	1.498	0.489	4.587	0.479
Coronary artery disease	0.911	0.389	2.135	0.831
Peripheral artery disease	1.664	0.146	18.935	0.682
SSS	1.065	0.507	2.237	0.869
Prior AF	15.484	5.408	44.339	<0.001
**Pharmacologic therapy**				
Beta-blockers	2.343	1.091	5.031	0.029
ACEi/ARB	2.093	0.910	4.813	0.082
Calcium channel blockers	0.877	0.412	1.865	0.733
Diuretics	1.011	0.473	2.163	0.977
Antiarrhythmic agents	17.176	5.705	51.715	<0.001
**Echocardiographic parameters**				
LVEF (%)	0.976	0.928	1.027	0.344
LVEDV, ml	1.003	0.984	1.023	0.740
LVESV, ml	1.039	0.990	1.090	0.119
LVMI, g/m^2^	1.002	0.991	1.014	0.700
LA dimension, cm	1.022	0.917	1.139	0.697
MV E, cm/s	1.002	0.982	1.021	0.866
MV A, cm/s	0.979	0.965	0.994	0.005
MV deceleration time, s	0.074	0.000	41.526	0.420
**OVFI group**				
Unadjusted GEE model				
20 < OVFI vs. OVFI ≥ 20	1.975	1.020	3.825	0.044
40 <OVFI vs. OVFI ≥ 40	2.469	1.202	5.071	0.014
60 <OVFI vs. OVFI ≥ 60	1.567	0.654	3.756	0.314
Adjusted GEE model *				
20 <OVFI vs. OVFI ≥ 20	2.294	1.061	4.961	0.035
40 <OVFI vs. OVFI ≥ 40	2.419	0.954	6.135	0.063
60 <OVFI vs. OVFI ≥ 60	1.710	0.708	4.129	0.233

* Adjusted for age, sex, prior AF, prior CHF, use of anti-arrhythmic agents and beta-blockers, and MV A. Covariates were identified using backward selection, and *p* values < 0.10 were retained in the model. ACEi, angiotensin-converting enzyme inhibitor; AF, atrial fibrillation; ARB, angiotensin receptor blockers; BMI, body mass index; CVA, cerebrovascular accident; GEE, generalized estimating equations; LA, left atrium; LVEDV, left ventricular end-diastolic volume; LVEF, left ventricular ejection fraction; LVESV, left ventricular end-systolic volume; LVMI, left ventricular mass index; MV, mitral valve; OVFI, OptiVol fluid index; SSS, sick sinus syndrome; TIA, transient ischemic attack.

**Table 4:** AT/AF burden, incidence, and follow-up duration in the two OVFI groups.

	20 < OVFI	OVFI ≥ 20	*p* Value
Number of visits	92	119	
With AT/AF ≥ 1 min	37	57	0.165
With AT/AF ≥ 10 min	23	41	0.091
With AT/AF ≥ 1 h	23	34	0.337
With AT/AF ≥ 4 h	17	33	0.079
With AT/AF ≥ 24 h	11	15	0.530
AT/AF burdens, %	9.0 ± 22.3%	7.4 ± 21.8%	0.612
AT/AF episodes			
≥1 min per day	81.7 ± 296.6	268.7 ± 1065.8	0.104
≥10 min per day	8.0 ± 39.1	18.6 ± 87.0	0.241
≥1 h per day	3.6 ± 15.9	6.7 ± 25.6	0.282
≥4 h per day	1.7 ± 6.9	8.0 ± 26.5	0.015
4–24 h per day	1.4 ± 6.6	6.0 ± 18.0	0.011
≥24 h per day	0.3 ± 1.1	2.0 ± 11.4	0.124
Follow-up duration, h	830.3 ± 1191.7	3716.2 ± 1804.2	<0.001

AF, atrial fibrillation; AT, atrial tachyarrhythmia; OVFI, OptiVol fluid index.
